# Pharmacokinetics of Ginkgolide B after Oral Administration of Three Different Ginkgolide B Formulations in Beagle Dogs

**DOI:** 10.3390/molecules201119678

**Published:** 2015-11-06

**Authors:** Jie Zhao, Ting Geng, Qi Wang, Haihong Si, Xiaoping Sun, Qingming Guo, Yanjing Li, Wenzhe Huang, Gang Ding, Wei Xiao

**Affiliations:** 1 State Key Laboratory of Pharmaceutical New-Tech for Chinese Medicine, Jiangsu Kanion Pharmaceutical Co., Ltd., Lianyungang 222000, China; zhaojiecpu@163.com (J.Z.); yilinger110@126.com (T.G.); sihaihong@163.com (H.S.); sunxiaoping721@163.com (X.S.); guoqingmgmg7380@soho.com (Q.G.); yanjinglicpu@126.com (Y.L.); njhwzh@hotmail.com (W.H.); dingg2000@126.com (G.D.); 2 Department of Pharmaceutics, Shenyang Pharmaceutical University, Shenyang 110016, China; kywangqi@126.com

**Keywords:** ginkgolide B, hot-melt extrusion, liquid layer, beagle dog plasma, pharmacokinetics, LC-MS/MS

## Abstract

Ginkgolide B (GB), an important active constituent of *Ginkgo biloba* extract, has been used in clinical applications for the treatment of dementia, cerebral insufficiency or related cognitive decline. To investigate the main pharmacokinetic characteristics of three different GB formulations in beagle dogs, a simple, specific and sensitive LC-MS/MS method was established and validated. The separation of the analytes was achieved on an Agilent Eclipse Plus C_18_ column (1.8 μm, 2.1 × 50 mm) with a mobile phase consisting of water and acetonitrile. The flow rate was set at 0.4 mL/min. Quantitation was performed using multiple reaction monitoring (MRM) in negative ion mode, with the transitions at *m*/*z* (Q1/Q3) 423.1/367.1 for GB and *m*/*z* 269.3/170.0 for IS. The linear calibration curve of GB was obtained over the concentration range of 2–200 ng/mL. The intra- and inter-day precisions were <15% and the accuracies were within ±12.7%. The validated method was applied to compare the pharmacokinetic characteristics of GB in healthy beagle dogs after oral administration of three formulations (HME08, GB capsule prepared by hot-melt extrusion technology; LL06, GB pellet prepared by liquid layer technology; conventional GB tablet). The C_max_ values of GB from different formulations in beagle dog plasma were 309.2, 192.4 and 66.6 µg/L, and the AUC values were 606.7, 419.1 and 236.2 µg/L·h, respectively. The data suggested that the exposure level of GB from HME08 and LL06 in beagle dog plasma was greatly improved compared with conventional tablets. This study should be helpful for the design and development of oral GB preparations.

## 1. Introduction

**Figure 1:**
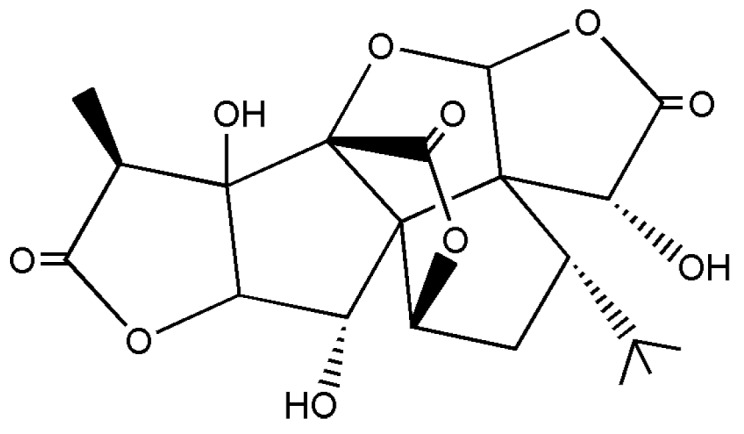
The chemical structure of GB.

Ginkgo biloba extract (GBE), an extract of *Ginkgo biloba* tree leaves, has been used in clinic for the treatment of vascular and age-related deterioration of cognitive functions, including dementia and peripheral arterial occlusive diseases [1,2]. Previous studies indicated that terpene lactones—the ginkgolides and bilobalide—as well as flavonol glycosides, are the main contributors to the medical effects of the extract [3,4]. Particularly, accumulating evidence suggests that ginkgolide B (GB, chemical structure shown in Figure 1) exhibited the strongest pharmacological activity among the ginkgolides. It could reduce the platelet aggregation and the formation of thrombosis by selectively and effectively antagonizing the platelet activating factor [5]. However, the wide use of GB has been limited due to its poor water solubility and the low oral bioavailability and limited the desired effect of marketed products, so it is necessary to develop a new formulation of GB to increase the oral absorption and bioavailability.

There are a number of formulation strategies for the poorly soluble drugs. In recent years, several approaches have been developed for enhancing the solubility and oral bioavailability of GBE, such as solid dispersions [6,7], self-emulsification [8,9,10,11], phospholipid complexation [12,13] and so on. In this paper, to combat the unsatisfactory bioavailability of GB, two new GB formulations prepared by hot-melt extrusion (HME) and liquid layer technology are evaluated. Hot-melt extrusion technology, extensively used in the plastics industry for more than fifty years, is arousing increasing interest in the pharmaceutical industry for the manufacture of drug delivery systems [14]. It has been proved that compounds with poor bioavailability due to their solubility limitations would be prime candidates for HME, by which the active components are embedded in a carrier system, usually comprised of one or more thermoplastic polymers and/or melting waxes [15,16]. Pellets prepared by liquid layer technology are also successful in improving bioavailability according to previous studies. Pellets greatly increases the interaction surface between drugs and the stomach or intestine compared with conventional tablets [17,18]. While selecting and optimizing the accessories as well as technological parameters, we acquired three GB formulations, named HME08, LL06 and conventional tablets, respectively. Moreover, the subsequent *in vitro* experiments revealed that HME08 and LL06 had good dissolution compared with conventional tablet. However, the desirable results gained from *in vitro* experiments do not mean excellent exposure of GB from HME08 and LL06 *in vivo*. Thus, determination of the preclinical pharmacokinetic profile of the three GB formulations is necessary and important.

In recent years, well-developed analytical methods, especially liquid chromatography-mass spectrometry, were widely used for the pharmacokinetic study of ginkgolides in animals and humans because of its high sensitivity and specificity. Huang *et al.* [19] and Xie *et al.* [20] successfully used a LC-MS/MS method to study the pharmacokinetics of terpene lactones. Lv *et al.* [21] established a sensitive and selective LC-MS method for the determination of pure GB in dog plasma. However, to our knowledge, there has been no research on the comparative pharmacokinetics of GB in beagle dogs after the conventional formulations were improved by some advanced technologies. In the present paper, a simple, specific and sensitive LC-MS/MS method was established to evaluate the pharmacokinetic characteristics of GB after oral administration of three different GB formulations in beagle dogs, which could provide a basis for the design and development of clinical oral GB preparations in future studies.

## 2. Experimental

### 2.1. Chemicals and Reagents

Ginkgolide B (GB) was purchased from National Institute for Drug and Food Control (Nanjing, China). Tolbutamide (internal standard, IS) was obtained from Aladdin Co. Ltd. (Shanghai, China). All compounds were of 99% purity. Methanol and acetonitrile of HPLC grade were purchased from Tedia Co. Ltd. (Farfield, OH, USA). Water was purified through a Milli-Q UV plus system. Three GB formulations were prepared by Shenyang Pharmaceutical University, as follows: HME08 was prepared by hot-melt extrusion technology, and each capsule contained 50 mg GB; LL06 was made by liquid layer technology, and each pellet contained 100 mg GB; each conventional GB tablet contained 100 mg GB.

### 2.2. Animals

The male beagle dogs (15.0 ± 0.1 kg) were purchased from the Shanghai Sino-British Sippr/BK LAB Animal Co. Ltd. (Shanghai, China). All animals were maintained in a normally controlled breeding room (temperature: 22 ± 2 °C, humidity: 50% ± 5%, 12 h dark/light cycle) with standardized diet and acclimatized for 7 days prior to the experiments. The beagle dogs were fasted overnight (12 h) before dosing with free access to water. All animal experiments were approved by the Kanion Co. Ltd. Animal Ethics Committee (Jiangsu, China).

### 2.3. Instrumentation and LC-MS/MS Conditions

#### 2.3.1. Chromatographic Conditions

Chromatographic analysis was performed on a Nexera X2 Series LC system (Shimadzu, Kyoto City, Japan) consisting of a binary pump (LC-30AD), a SIL-30AC autosampler and a CTO-30A column oven. Chromatographic separation was achieved on an Eclipse Plus C_18_ column (1.8 μm, 2.1 × 50 mm, Agilent, Foster City, CA, USA) at a flow rate of 0.4 mL/min. The mobile phase was the mixture of water (A) and acetonitrile (B). The elution gradient program was as follows: (time (min), (% mobile phase B): (0, 20) (1.5, 30) (3.0, 90) (3.1, 20) (4.5, 20)). The column was maintained at 25 °C and the injection volume was 4 μL.

#### 2.3.2. Mass Spectrometry Conditions

Mass spectrometry was performed on an API4000^+^ triple quadrupole mass spectrometer from Applied Biosystems (AB SCIEX, Foster City, CA, USA), equipped with a Turboionspray™ source in negative ion mode. The parameters in the source were set as follows: ion spray voltage −4.5 kV, ion-source temperature 500 °C, nebulizer gas (gas1) 50 psi; heater gas (gas2) 50 psi; curtain gas 25 psi; collision gas 6 psi. Quantitation of compounds were achieved by multiple reaction monitoring (MRM) mode, with the transition of *m*/*z* (Q1/Q3) 423.1/367.1 for GB and *m*/*z* (Q1/Q3) 269.3/170.0 for IS. The collision energy (CE) −20 eV, declustering potential (DP) −90 V and collision cell exit potential (CXP) −13 V for GB and IS. All the analytical data were processed by the Analyst software (version 1.6.1).

### 2.4. Plasma Sample Preparation

The plasma samples were kept at −80 °C and thawed at room temperature in 30 min prior to processing. An aliquot of 100 μL plasma sample was added to 800 μL ethyl acetate containing 15 ng/mL IS and vortex-mixed. After centrifugation at 12,000 rpm for 5 min, 700 μL of the supernatant was collected and evaporated to dryness at 40 °C under a gentle stream of nitrogen. Then the residue was reconstituted with 100 μL acetonitrile and centrifuged at 12,000 rpm for 3 min. 4 μL of the supernatant was injected into the LC-MS/MS system for analysis.

### 2.5. Preparation of Stock and Sample Solutions

A stock solution of GB was prepared by dissolving the accurately weighed reference compound in methanol at the concentration of 1 mg/mL. Then the solution was serially diluted with methanol to achieve standard working solutions at the concentrations of 20, 40, 100, 200, 500, 1000 and 2000 ng/mL for GB. A 15 ng/mL IS working solution was prepared by diluting the 0.1 mg/mL stock solution of tolbutamide with ethyl acetate.

### 2.6. Method Validation

The LC-MS/MS method was validated according to the US Food and Drug Administration guidelines on bioanalytical method validation [22].

#### 2.6.1. Specificity

The specificity was assessed by comparing chromatograms of blank plasma from six different beagle dogs, blank plasma extracted by ethyl acetate containing IS, blank plasma spiked with GB (LLOQ, 2 ng/mL), and a plasma sample collected at 1 h after a single oral administration of conventional GB tablet to a beagle dog.

#### 2.6.2. Linearity and Lower Limit of Quantitation

The calibration plasma samples were prepared by adding 10 μL working solution into 90 μL pooled blank beagle dog plasma, and then the samples were treated in accordance with the “plasma sample preparation”. The calibration samples were set at 2, 4, 10, 20, 50, 100 and 200 ng/mL. Calibration curve (peak area ratios of GB to IS against the nominal concentrations) was fitted by least-square linear regression using 1/x^2^ as the weighting factor. The correlation coefficient should be >0.9900. The lower limit of quantitation (LLOQ) was defined as the lowest concentration of GB on the calibration curve. The accepted criteria for the back-calculated standard concentrations was that they were within ±15% except for the LLOQ (2 ng/mL), which was set within ±20% (*n* = 6).

#### 2.6.3. Accuracy and Precision

The inter- and intra-day precisions were performed at three different QC levels (5, 80 and 160 ng/mL, *n* = 6) on the same day and three consecutive days. The precisions were evaluated by relative standard deviation (R.S.D), which should be <15%. The accuracies were assessed by comparing the calculated concentration with the nominal concentration, and should be within 85%–115%.

#### 2.6.4. Extraction Recovery and Matrix Effect

The extraction efficiency of GB in beagle dog plasma was investigated by determining the response ratios of analytes in post-extraction spiked QC samples to that obtained from pre-extraction spiked samples. The matrix effect was evaluated by comparing the response of analyte spiked in pretreated blank plasma sample with that obtained from pretreated methanol at equivalent concentrations. The results of extraction recovery and matrix effect should be stable and repeatable.

#### 2.6.5. Stability

The stability experiments of GB in beagle dog plasmas were explored by analyzing replicates (*n* = 4) of plasma samples at LQC (5 ng/mL) and HQC (160 ng/mL) levels under the following conditions: bench-top stability (room temperature for 6 h), post-preparative stability (4 °C for 24 h), long-term stability (−80 °C for about 30 days) and freeze-thaw stability (−80 °C–25 °C for three cycles). Samples were considered stable if the accuracies of samples were within 15% at different levels, while the precisions should not exceed 15%.

#### 2.6.6. Dilution Effect

Dilution effect was investigated by spiking the known standard solution in blank beagle dog plasma which far exceeded the upper limit of quantitation (ULOQ). Then the QC samples were diluted by blank plasma and the dilution factor was 10. The diluted samples were treated according to “plasma sample preparation”. The acceptable accuracy and precision should not exceed 15% for the QC samples.

### 2.7. Pharmacokinetic Study

Fifteen male beagle dogs were randomly divided into three groups: HME08, LL06 and conventional GB tablet. Then the beagle dogs were orally administrated with three GB formulations at the dosage of 20 mg/kg (six HME08 capsules, three LL06 pellets and three conventional tablets). About 500 μL blood samples were collected according to the specific schedule, 0, 0.33, 0.66, 1, 2, 3, 4, 6, 8, 10, 12, 14, 16, 24 h after the treatment of GB preparations. All blood samples were put into heparinized micro-centrifuge tubes and followed by centrifuging at approximately 12,000 rpm for 3 min. The resulting plasma layers were transferred into clean tubes immediately and maintained at −80 °C until LC-MS/MS analysis performed with the procedure described above.

### 2.8. Statistical Analysis

Data acquisition and peak integration were performed using Analyst software (version 1.6.1). Pharmacokinetic parameters were calculated by DAS 2.1 software (Mathematical Pharmacology Professional Committee of China, Shanghai, China) according to non-compartmental model. Statistical significance was assessed by an unpaired Student’s t-test and the significance level of *p* < 0.05 was adopted for all statistical comparisons.

## 3. Results and Discussion

### 3.1. MS/MS Optimization

In negative ESI mode, GB and IS exhibited de-protonated molecules [M − H]^−^ at *m*/*z* 423.1 and 269.3, respectively. The specific product ions with *m*/*z* values of 367.1 (GB) and 170.0 (IS) were selected as target ions for quantitative analysis.

### 3.2. Sample Preparation

Liquid-liquid extraction and protein precipitation were two common plasma treatment methods in pharmacokinetic studies. Peak shape, matrix effect and recovery were under consideration for evaluating which method more suitable in this study. When methanol and acetonitrile were tested for protein precipitation, low extraction recoveries were observed (data not shown). Next we selected ethyl ether and ethyl acetate as liquid-liquid extraction solvents. Finally, ethyl acetate was selected as the extraction solvent because of its high and stable recovery efficiency as well as perfect peak shape.

### 3.3. Method Validation

#### 3.3.1. Specificity

**Figure 2:**
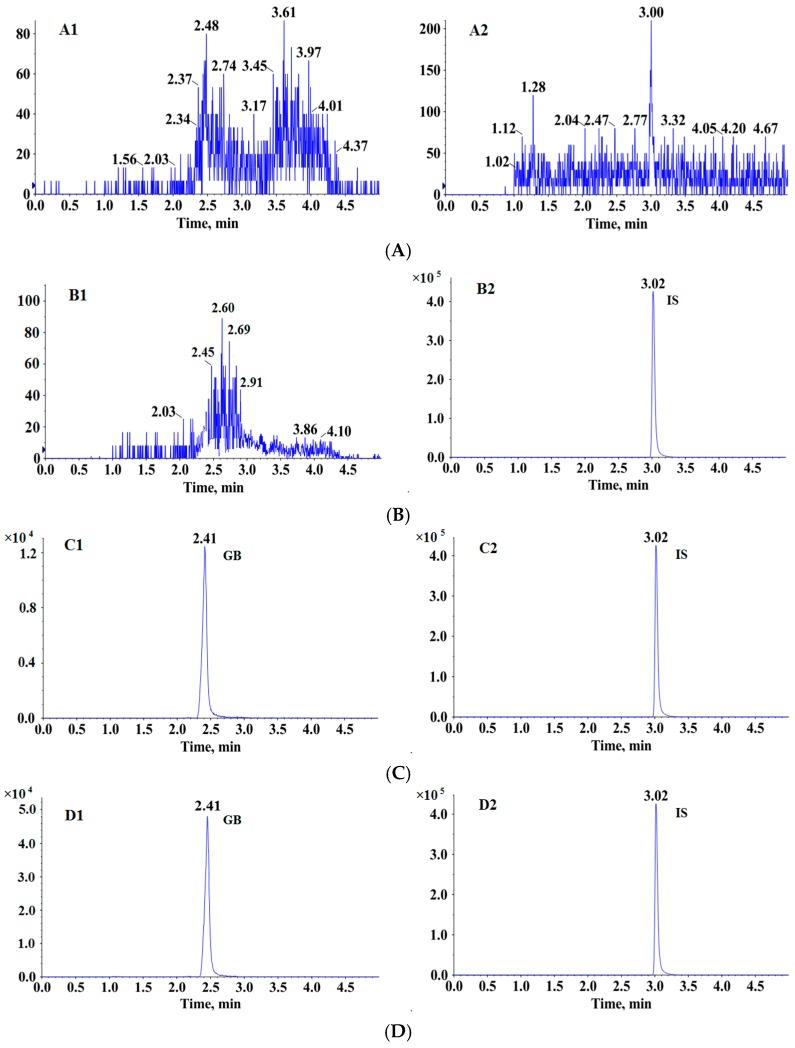
Representative MRM chromatograms of GB and IS in beagle dog plasma. (**A**) blank beagle dog plasma; (**B**) blank beagle dog plasma extracted by ethyl acetate containing IS (15 ng/mL); (**C**) blank beagle dog plasma spiked with 2 ng/mL GB (LLOQ) and IS; (**D**) plasma sample collected at 1 h after a single oral administration of conventional GB tablet to a beagle dog. (1, MRM of channels ESI^−^ 423.1/367.1 for GB; 2, MRM of channels ESI^−^ 269.3/170.0 for IS).

Six blank beagle dog plasma samples (Figure 2A), blank beagle dog plasma extracted by ethyl acetate containing IS (Figure 2B), blank beagle dog plasma spiked with GB (LLOQ, 2 ng/mL) (Figure 2C), and a plasma sample collected at 1 h after a single oral administration of conventional GB tablet to a beagle dog (Figure 2D) were assessed. GB and IS were well separated from the beagle dog plasma matrix components under the described chromatographic conditions with the retention times at 2.4 and 3.0 min, respectively. The signal observed on the MRM chromatogram of all blank beagle dog plasma samples was less than 20% of those acquired in the LLOQ samples (*n* = 6). Notably, area response of GB from the blank plasma sample extracted by ethyl acetate containing IS (15 ng/mL) was similar to the blank beagle dog plasma sample, indicating that IS didn’t contribute to GB signal. In addition, GB didn’t contribute to the IS response. No endogenous interfering peaks were observed at or near the retention time of GB and IS by comparing the chromatograms. Consequently, these results suggested our method had high selectivity for both GB and IS.

#### 3.3.2. Linearity and LLOQ

**Figure 3:**
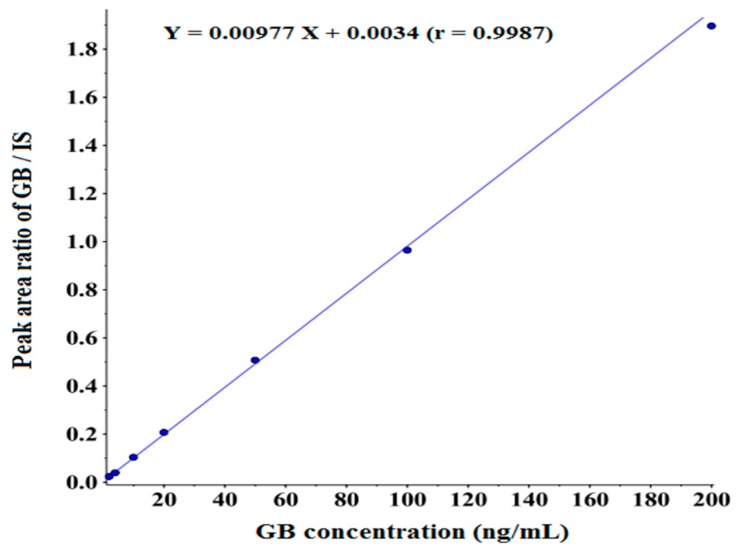
Linear regression calibration curve of GB in beagle dog plasma.

The calibration curve of GB in beagle dog plasma was constructed by plotting peak area ratios of GB/IS versus the concentrations of GB using the weighted (1/x^2^) least squares linear regression. The method showed good linearity over the concentrations range from 2 to 200 ng/mL with a correlation coefficient (r) >0.9900. The typical calibration curve was presented in Figure 3. The lower limit of quantitation meeting the acceptance criteria of ±20% and <20% in terms of accuracy and precision was 2 ng/mL and data was shown in Table 1. The method proved to be sensitive enough for the determination of GB in beagle dog plasma.

#### 3.3.3. Accuracy and Precision

The obtained inter- and intra-day accuracies and precisions were summarized in Table 1. The intra-day accuracies ranged from 103.4% to 112.7%, along with precisions ranged from 1.8% to 9.4%. The inter-day accuracies changed from 94.6% to 103.5%, while precisions ranged from 4.6% to 8.6%. All the values were within the acceptable range.

**Table 1:** Summary of accuracy and precision for the determination of GB in beagle dog plasma.

Concentration (ng/mL)	Intra-Day (*n* = 6)			Inter-Day (*n* = 18)		
	Mean (95% Cl) (ng/mL)	RSD (%)	Accuracy (%)	Mean (95% Cl) (ng/mL)	RSD (%)	Accuracy (%)
2 (LLOQ)	1.99 (1.93, 2.05)	3.9	99.3			
5 (QCL)	5.17 (4.77, 5.57)	9.4	103.4	4.73 (4.54, 4.92)	8.6	94.6
80 (QCM)	90.15 (87.56, 92.74)	3.6	112.7	82.79 (79.56, 86.02)	8.4	103.5
160 (QCH)	173.7 (171.2, 176.1)	1.8	108.5	165.1 (161.6, 168.5)	4.6	103.2

#### 3.3.4. Matrix Effect and Recovery

Table 2 listed the extraction recovery and matrix effect of GB in bio-samples. The extraction recoveries of GB at three QC levels were 82.09%, 84.16% and 81.16%, respectively. These results indicated the recoveries of GB were consistent and reproducible at different levels in beagle dog plasma. For an LC-MS/MS method, it was imperative to investigate the matrix effect on analyte ionization caused by co-eluting components. The matrix effects at three QC levels were 105.7%, 99.47% and 99.70% for GB, respectively. In addition, the matrix effect and recovery of IS were investigated and the recoveries ranged from 91.40% to 114.0%, along with matrix effects ranged from 85.52% to 91.61%. Thus, no significant matrix effect was found for GB and IS, which indicated that the co-eluting substances had no obvious influence on the ionization of analytes.

**Table 2:** Extraction recovery and matrix effect for GB in beagle dog plasma (*n* = 6).

Concentration (ng/mL)	Extraction Recovery		Matrix Effect	
	Mean ± SD (%)	RSD (%)	Mean ± SD (%)	RSD (%)
5 (QCL)	82.09 ± 3.05	3.7	105.7 ± 2.51	2.4
80 (QCM)	84.16 ± 3.24	2.1	99.47 ± 3.37	3.4
160 (QCH)	81.16 ± 1.65	2.0	99.70 ± 2.07	2.1

#### 3.3.5. Stability

A summary of stability evaluation of GB was given in Table 3. It could been seen that GB in beagle dog plasma could remain stable under the following treatment: −80 °C–25 °C for three cycles (freeze-thaw stability), −80 °C for 30 d (long-term stability) and 4 °C for 24 h (post-preparative stability). The back-calculated concentrations of these samples were comparable to the nominal values, with accuracies ranging from 93.63% to 104.6%. However, it was noteworthy that a significant degradation of GB was observed after maintaining at room temperature for 6 h. Next we investigated the room temperature at 1, 2, 3, 4, 5 h, respectively, and found that GB in beagle dog plasma was of good stability within 4 h. It was speculated that the hydrolyzed form of GB was formed in bio-samples at room temperature [23]. These results indicate that plasma samples should be kept in −80 °C and treated in an ice bath and as quickly as possible in the case of potential GB degradation.

**Table 3:** Stability of GB in beagle dog plasma (*n* = 4).

Sample Condition	Concentration (ng/mL)	Mean ± SD (ng/mL)	RSD (%)	Accuracy (%)
bench-top stability	5	0.53 ± 1.06	/	11.6
	160	15.08 ± 1.23	8.1	9.43
post-preparative stability	5	5.23 ± 0.48	9.1	104.6
	160	149.8 ± 6.29	4.2	93.63
freeze –thaw stability	5	5.19 ± 0.50	9.6	103.9
	160	163.8 ± 11.03	6.7	102.3
long-term stability	5	4.69 ± 0.61	13.1	93.80
	160	151.5 ± 5.92	3.9	94.69

“/” not available.

#### 3.3.6. Dilution Effect

A high QC sample at a concentration of 400 ng/mL was diluted 10-fold with blank plasma prior to sample treatment and determined in five replicates together with the calibration and QCs in a validation run. As can be seen in Table 4, the determined concentrations of GB in these QCs were comparable to the nominal values, with an accuracy of 106.9%, implying that samples at higher concentrations could be reanalyzed by simple dilution.

**Table 4:** Sample dilution accuracy and precision (*n* = 6).

Concentration (ng/mL)	Mean ± SD (ng/mL)	RSD (%)	Accuracy (%)
400	427.5 ± 32.12	7.5	106.9

### 3.4. Pharmacokinetic Study

**Figure 4:**
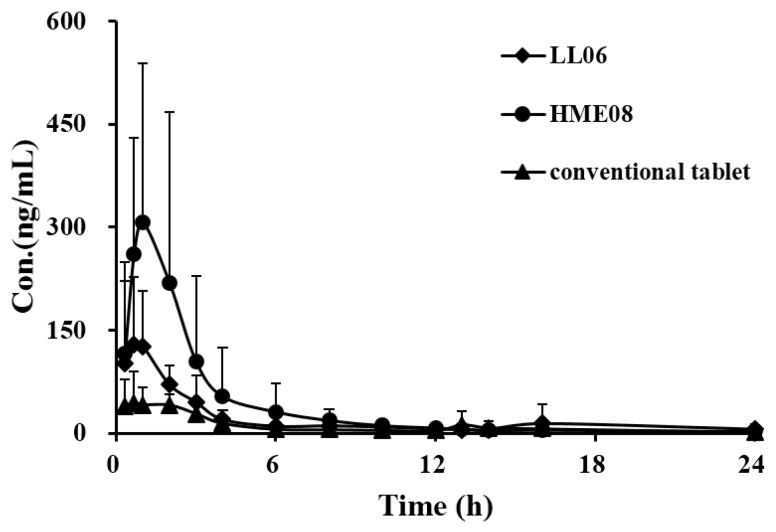
Mean plasma concentration-time profiles of GB from three GB formulations (conventional tablet, HME08, and LL06) in beagle dog after oral administration at the dosage of 20 mg/kg (*n* = 5).

A summary of the pharmacokinetic parameters for GB from HME08, LL06 and conventional tablet in beagle dogs after oral administration at the dose of 20 mg/kg was given in Table 5, and the mean plasma concentration-time profile of GB was presented in Figure 4. Following the oral administration of HME08 or LL06, the maximum concentrations (C_max_) were achieved after roughly 1 h. In contrast, the highest GB plasma concentration for conventional GB tablet was attained at approximately 3.7 h. It was clear that the peak concentrations of GB in beagle dog plasma after treatment with HME08 or LL06 was significantly increased compared with conventional GB tablets. Though C_max_ of GB in the HME08 group was slightly higher than that in the LL06 group, they didn’t show a statistical difference (*p* > 0.05). No significant differences were observed in the parameters of t_1/2z_ and mean residence time (MRT) among all three GB formulations. It is noteworthy that the areas under curves (AUC_0-t_) were 606.74 ± 125.03 and 419.12 ± 55.58 µg/L·h for HME08 and LL06, but only 236.16 ± 81.14 µg/L·h for conventional tablets. Unlike the maximum concentration, a significant difference was found between the AUC values of HME08 and LL06. The above results indicate that HME08 and LL06 promoted the absorption of GB in beagle dogs, while had no obvious influences on the elimination, thus resulting in the stable AUC and invariable t_1/2_. In addition, it is known that the exposure to GB is critical for its medical effects, so we speculate that HME08 will exhibit the best pharmacological effects among these three GB preparations.

**Table 5:** Pharmacokinetic parameters of GB in beagle dog plasma after oral administration of conventional GB tablet, HME08 and LL06 (*n* = 5).

Parameters	Conventional Tablet	HME08	LL06
AUC_0-t_ (µg/L·h)	236.2 ± 81.14	606.7 ± 125.03 *^,#^	419.1 ± 55.58 *
AUC_0-∞_ (µg/L·h)	246.8 ± 92.08	672.0 ± 59.89 *^,#^	438.9 ± 71.14 *
MRT_0-t_ (h)	5.65 ± 3.42	4.18 ± 1.55	3.53 ± 1.77
MRT_0-∞_ (h)	6.58 ± 3.54	8.75 ± 7.63	4.30 ± 2.38
t_1/2z_ (h)	4.45 ± 3.04	4.14 ± 1.81	3.14 ± 1.80
T_max_ (h)	3.73 ± 1.21	0.80 ± 0.18	1.20 ± 1.04
CLz/F (L/h/kg)	90.94 ± 33.97	29.96 ± 2.70	46.68 ± 8.65
Vz/F (L/kg)	560.2 ± 405.4	405.7 ± 398.5	216.1 ± 132.4
C_max_ (µg/L)	66.64 ± 29.30	309.2 ± 106.0 *	192.4 ± 84.22 *

Values are mean ± SD; SD, standard deviation; AUC_0-t_, area under the concentrations-time curve; AUC_0-∞_, area under the curve with extrapolation to infinity; t_1/2z_, elimination half-life; C_max_, maximum plasma concentration, T_max_, time to peak; Vz, apparent volume of distribution; CL, clearance; MRT, mean residence time; * *p* < 0.05 when compared with the level of corresponding conventional tablet; ^#^ *p* < 0.05 when compared with the level of corresponding LL06.

HME offers several advantages over traditional processing techniques for pharmaceutical applications. The process is anhydrous, entails a continuous operation necessitating fewer processing steps, requires no compression of the actives and improves the bioavailability due to the dispersion of drugs at the molecular level in the final dosage forms [24,25]. Several products prepared by HME technology have been approved in the US or Asia, which suggests that this emerging technique is being gradually recognized by the public. In our study, improved oral bioavailability of GB in beagle dogs has also revealed the value of HME as a powerful drug delivery tool. Although the high temperature employed and the shear forces generated during HME processing may result in drug degradation, these shortages may be overcome by appropriate formulations and equipment design as well as engineering approaches [26].

## 4. Conclusions

In summary, a highly selective and simple LC-MS/MS method was established and validated for the determination of GB in beagle dog plasma. The method was reliable and showed highly reproducible chromatographic and statistical results in terms of precision and accuracy during the validation. The method was successfully applied to the comparative pharmacokinetic study of three GB formulations in beagle dogs. Based on the results of pharmacokinetic study, it could be concluded that there was significant difference in the pharmacokinetic characteristics of GB among the three preparations. This study indicated that the application of hot-melt excursion or liquid layer technology could greatly improve the exposure of GB in beagle dogs compared with conventional GB tablets. In addition, our study lays the foundation for the design and development of novel oral GB preparations in future studies.
