# Correction: Zhao, J., *et al.* Pharmacokinetics of Ginkgolide B after Oral Administration of Three Different Ginkgolide B Formulations in Beagle Dogs. *Molecules* 2015, *20*, 20031–20041

**DOI:** 10.3390/molecules21060759

**Published:** 2016-06-09

**Authors:** Jie Zhao, Ting Geng, Qi Wang, Haihong Si, Xiaoping Sun, Qingming Guo, Yanjing Li, Wenzhe Huang, Gang Ding, Wei Xiao

**Affiliations:** 1 State Key Laboratory of Pharmaceutical New-Tech for Chinese Medicine, Jiangsu Kanion Pharmaceutical Co., Ltd., Lianyungang 222000, China; zhaojiecpu@163.com (J.Z.); yilinger110@126.com (T.G.); sihaihong@163.com (H.S.); sunxiaoping721@163.com (X.S.); guoqingmgmg7380@soho.com (Q.G.); yanjinglicpu@126.com (Y.L.); njhwzh@hotmail.com (W.H.); dingg2000@126.com (G.D.); 2 Department of Pharmaceutics, Shenyang Pharmaceutical University, Shenyang 110016, China; kywangqi@126.com

The authors wish to correct the funding projects number of “Natural Science Foundation of Jiangsu Province” in the Acknowledgments section of this paper [1]: The correct funding projects number should be “No. BK20130403”, not “No. BK2013403”.

The authors would like to apologize for any inconvenience caused to the readers by this change.
